# Evidence of Dengue Virus Transmission and Factors Associated with the Presence of Anti-Dengue Virus Antibodies in Humans in Three Major Towns in Cameroon

**DOI:** 10.1371/journal.pntd.0002950

**Published:** 2014-07-10

**Authors:** Maurice Demanou, Régis Pouillot, Marc Grandadam, Pascal Boisier, Basile Kamgang, Jean Pierre Hervé, Christophe Rogier, Dominique Rousset, Christophe Paupy

**Affiliations:** 1 Virology Department, Centre Pasteur Cameroon, Member of the International Network of Pasteur Institutes (RIIP), Yaounde, Cameroon; 2 Epidemiology Department, Centre Pasteur Cameroon, Member of the International Network of Pasteur Institutes (RIIP), Yaounde, Cameroon; 3 Institut Pasteur du Laos, Laboratoire des Arbovirus et Maladies Virales Émergentes, Vientiane, Lao PDR; 4 Institut Pasteur de Bangui, Bangui, Central African Republic; 5 IRD, UR 016, Montpellier, France; 6 Institut Pasteur de Madagascar, Antananarivo, Madagascar; 7 Institut Pasteur de La Guyane, Laboratoire de Virologie, Cayenne, French Guiana; 8 UMR MIVEGEC (IRD 224 - CNRS 5290 - UM1 – UM2), Institut de Recherche pour le Développement, Montpellier, France; 9 Equipe Ecologie des Systèmes Vectoriels, Centre International de Recherches Médicales de Franceville, Franceville, Gabon; Institute of Collective Health, Federal University of Bahia, Brazil

## Abstract

**Background:**

Dengue is not well documented in Africa. In Cameroon, data are scarce, but dengue infection has been confirmed in humans. We conducted a study to document risk factors associated with anti-dengue virus Immunoglobulin G seropositivity in humans in three major towns in Cameroon.

**Methodology/Principal Findings:**

A cross sectional survey was conducted in Douala, Garoua and Yaounde, using a random cluster sampling design. Participants underwent a standardized interview and were blood sampled. Environmental and housing characteristics were recorded. Randomized houses were prospected to record all water containers, and immature stages of Aedes mosquitoes were collected. Sera were screened for anti-dengue virus IgG and IgM antibodies. Risk factors of seropositivity were tested using logistic regression methods with random effects.

Anti-dengue IgG were found from 61.4% of sera in Douala (n = 699), 24.2% in Garoua (n = 728) and 9.8% in Yaounde (n = 603). IgM were found from 0.3% of Douala samples, 0.1% of Garoua samples and 0.0% of Yaounde samples. Seroneutralization on randomly selected IgG positive sera showed that 72% (n = 100) in Douala, 80% (n = 94) in Garoua and 77% (n = 66) in Yaounde had antibodies specific for dengue virus serotype 2 (DENV-2).

Age, temporary house walls materials, having water-storage containers, old tires or toilets in the yard, having no TV, having no air conditioning and having travelled at least once outside the city were independently associated with anti-dengue IgG positivity in Douala. Age, having uncovered water containers, having no TV, not being born in Garoua and not breeding pigs were significant risk factors in Garoua. Recent history of malaria, having banana trees and stagnant water in the yard were independent risk factors in Yaounde.

**Conclusion/Significance:**

In this survey, most identified risk factors of dengue were related to housing conditions. Poverty and underdevelopment are central to the dengue epidemiology in Cameroon.

## Author Summary

General awareness of dengue fever in Africa, and particularly in Cameroon, is weak. Many acute febrile illnesses are considered as malaria, although not laboratory confirmed, and the diagnosis of dengue fever is seldom evoked while its laboratory confirmation is even more seldom obtained. On the basis of anti-dengue virus IgG seropositivity in humans, our survey demonstrated that dengue virus transmission occurred in the three main towns of the country. Although the findings varied according to the location, identified risk factors of anti-dengue virus seropositivity were commonly related to housing conditions. Taking into account the risk factors identified in Douala, practical ways to lower the risk of dengue virus transmission are long term development and improvement of sanitation and education. We concluded that poverty and underdevelopment are central to the problem of dengue virus transmission in urban areas in Cameroon.

## Introduction

Dengue fever is a mosquito-borne viral disease caused by four serologically distinct, but closely related, dengue viruses (DENV 1, 2, 3 and 4) belonging to the *Flaviviridae* family. DENV are transmitted from human to human by anthropophilic *Aedes* mosquitoes, mainly *Aedes aegypti* and *Aedes albopictus*. Dengue is found in tropical and sub-tropical climates worldwide, mostly in urban and semi-urban areas, and an estimated 2.5 billion people in more than 100 countries live in regions where the DENV are transmitted. More than 70% of the disease burden occurs in Asia and the Pacific, followed by the Americas, the Middle East and Africa [1]. The global incidence of dengue has sharply increased in recent decades and dengue fever is now considered the most important arthropod-borne viral disease of public health significance [2].

In Africa, dengue viruses do not give rise to large epidemics contrary to Asia, America or Pacific regions. It remains poorly documented in this continent. However, serological evidence of DENV infections has been established for years in several countries [3]–[8]. Close to Cameroon, Gabon experienced several DENV epidemics between 2007 and 2010 [9], [10], [11]. In Cameroon, where fever access are commonly attributed to malaria without any laboratory confirmation, data on dengue infection are scarce, although it has been confirmed in humans [12], [13], [14]. Entomological and virological surveys have shown that both the indigenous mosquito *Ae. aegypti* and the recently introduced mosquito *Ae. albopictus* were present in Cameroon [15], [16] and had a high vector competence for DENV [17].

Risk factors of DENV infection have been the subject of many surveys, particularly in South America and South East Asia. Most identified risk factors are those favorable to the vectors breeding, particularly unplanned and uncontrolled growth of urban centers associated with substandard housing, inadequate water supply system, malfunctioning waste management system in contexts of high density of dwelling [18], [19]. Therefore, the socio-economic dimension is a major character of DENV transmission. Behavioral risk factors have been documented, like water storage practices [20], while potential occupational risk factors of infection are still largely unknown. Individual risk factors like sex and race have been identified, but they appear to apply for susceptibility to disease rather than for infection [21], [22].

We report the findings of a survey of risk factors associated with the presence of anti-DENV IgG antibodies in humans in three major towns in Cameroon located in contrasting environment and climate.

## Methods

### Study sites

**Figure 1:**
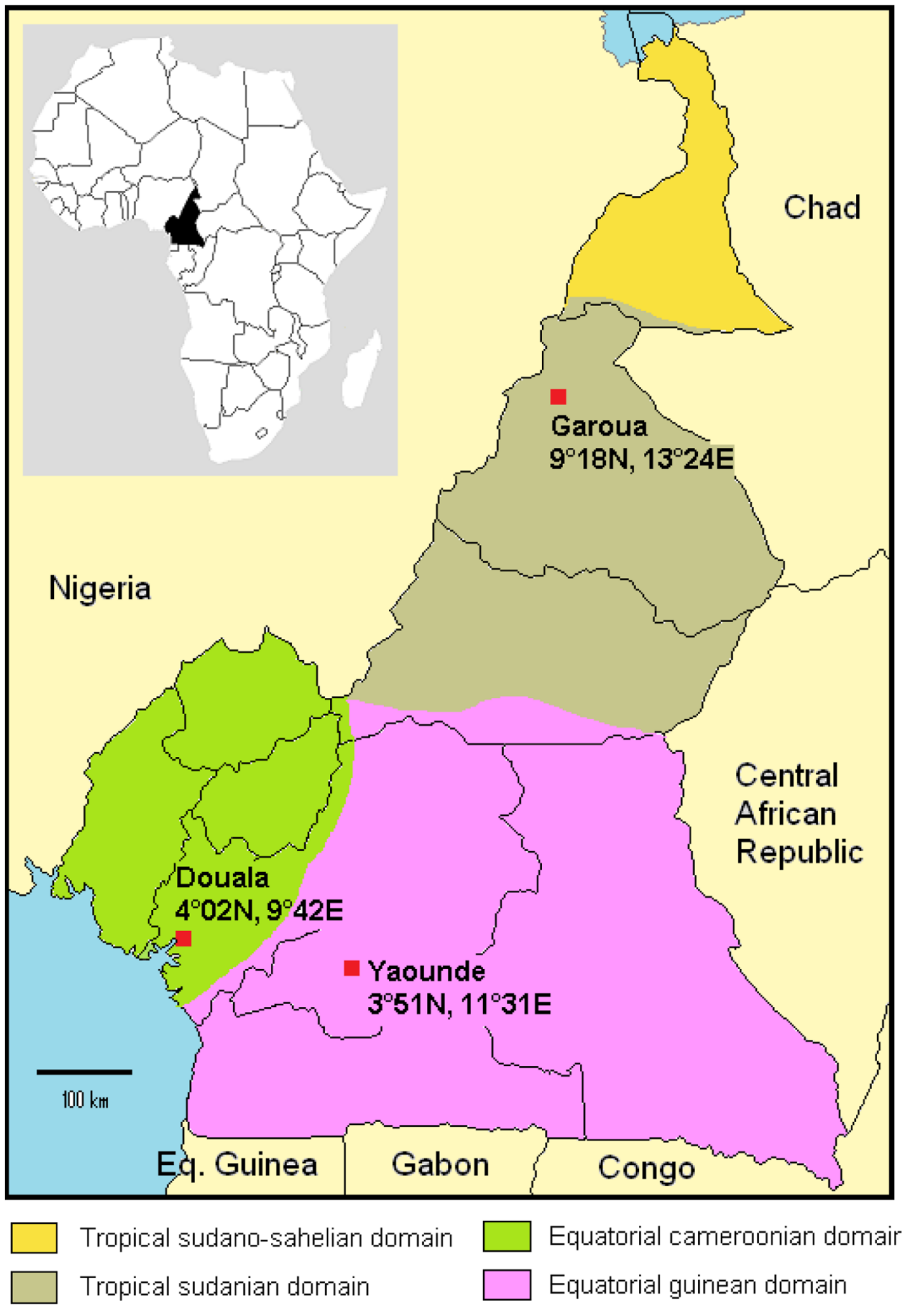
Map of Cameroon: main climatic zones and location of the 3 study sites.

The study was carried out in three major towns of Cameroon, settled in three different ecozones. Yaounde (3°51′N, 11°31′E) is the capital city, Douala (4°02′N, 9°42′E) is the economical capital and Garoua (9°18′N, 13°24′E) is a major town in the northern part of the country (Figure 1).

Yaounde is located 250 km inland at an altitude of 750 m. It had an estimated population of about 1.8 million in 2005. Yaounde receives an average 1600 mm rainfall annually, divided into 2 rainy seasons: the small one, peaking in May, and the main one, peaking in October. There is no dry season strictly speaking. Monthly mean air temperatures vary between 22°C and 25°C.

Douala is a coastal city of about 1.9 million inhabitants in 2005, located on the Atlantic coast, with an important harbor. Douala is a densely populated flat town where many districts, often resulting from unplanned development, suffer from absence of drainage and sewerage system. The climate of Douala is equatorial-monsoon. The average annual rainfall is 4000 mm and the rainiest months are between June and October. Monthly mean air temperatures vary between 24°C and 28°C.

Garoua had an estimated population of 236,000 inhabitants in 2005. The climate of Garoua is tropical-Sudanian, with a mean annual rainfall of 950 mm and a marked dry season from November to March. The monthly mean air temperatures vary between 26°C and 33°C.

The documentation of the geographic and ecological distribution of the dengue virus vectors *Ae. aegypti* and *Ae. albopictus* in these three towns was the matter of a previous publication [23].

### Study design

A cross sectional survey has been carried out in each town, using a random cluster sampling method based on cluster identification from geographical coordinates.

The sampling plan consisted in randomization of 75 GPS (Global Positioning System) points within each town, with probability proportional to the population density of districts according to the 3^rd^ General Population and Housing Census of Cameroon (3^rd^ GPHC, 2005). From each randomized point, the field investigators identified the three to five houses located around the GPS coordinates.

Persons living in selected households have been invited to participate. All participating individuals were interviewed using a standardized questionnaire form that was filled in by the investigator. Information that was common to all individuals belonging to the same household was recorded from the head of the family. Standardized environmental and housing characteristics were collected directly by the investigators, which were either epidemiologists or sociologists or geographers, depending on the nature of dataset to collect.

### Blood samples collection

A blood sample (5 ml) was collected in a Vacutainer tube (Becton Dickinson, Plymouth, UK) for all volunteers during household visit in each town, processed within 6 hours, and stored at −20°C. Sera were then transported to the Virology Laboratory of Centre Pasteur of Cameroon, in Yaounde, for serologic diagnosis.

### Serologic diagnosis

Sera were screened for IgM and IgG antibodies to DENV using “in-house” techniques: IgM capture enzyme immunoassay (MAC-ELISA) and ELISA IgG anti dengue virus (method used by the Institut de Médecine Tropicale du Service de Santé des Armées (IMTSSA), Marseille, France).

Briefly, goat anti-human IgM antibodies (Jackson ImmunoResearch Laboratories Inc., West Grove, USA) were passively adsorbed onto ELISA plates (Maxisorp, Nunc, Denmark) overnight at +4 C. After being washed four times in PBS/0.05% Tween20, 100 µl of serum diluted 1∶200 in PBS/Tween/3% Difco skim milk (Becton Dickinson, Le Pont de Claix, France) was added and incubated for 1 hour 30 minutes at 41°C. After washes, one hundred microliters of purified virus antigen prepared on Vero cells and inactivated by betapropiolactone (Sigma-Aldrich, St Quentin Fallavier, France) was applied to each well, and plates were incubated for 2 h at 41°C. Plates were then washed and covered with 100 µl/well of hyper immune ascitic fluid and incubated 1 hour at 41°C. Specific binding was demonstrated by using a peroxidase-labeled goat anti-mouse IgG conjugate (Jackson ImmunoResearch Laboratories Inc., West Grove, USA). After incubation 1 hour at 41°C and washing steps, 80 µl/well of the TMB (tetramethylbenzidine) substrate (Interchim, Montluçon, France) was added and plates were incubated at room temperature for 10 minutes for color development. The reaction was stopped by the addition of 50 µl/well of 1 M hydrochloric acid and the absorbance was read at 450 nm with a PR2100 Biorad plate reader.

For the detection of IgG, microplates were coated with goat anti-mouse IgG antibodies (Jackson ImmunoResearch Laboratories Inc., West Grove, USA) and a specific binding was demonstrated by using a peroxidase-labeled goat anti-human IgG conjugate (Jackson ImmunoResearch Laboratories Inc., West Grove, USA).

For the validity of the assays, the following ratio (R) was calculated: optical density (OD) of the positive control or tested sera/OD of the negative control. Serum samples were considered positive if R≥3, negative if R<2.5 and equivocal if 2.5<R<3.

Because of the antigenic cross-reactivity among viruses of the *Flavivirus* genus, we performed a confirmation on a randomly selected sample of ELISA IgG positive sera, using a plaque reduction neutralization test (PRNT), according to De Madrid & Porterfield method [24]. Although laborious and time consuming, PRNT is considered the serologic reference method. Briefly, in a 96-well plate, four dilutions of each serum sample (1∶20, 1∶40, 1∶80, 1∶160) were incubated at 37°C for 1 hour in a viral suspension of DENV (1, 2, 3 and 4) and tick borne encephalitis virus (TBEV) of 10–50 PFU in 50 µL before the addition of 100 µL of a Vero cell suspension. Four days later, the cell layer was fixed in formol and stained with crystal violet. The assay run was considered valid only if the following criteria were met: average plaque count for virus control wells fell within the required target; the cell control wells showed no plaques; the titer of the positive assay quality control serum sample was within the pre-established acceptable ranges; and the titer of the negative assay quality control serum sample remained negative. IgG in sera which yielded negative result for DENV-1, -2, -3 and -4, and TBEV using PRNT were considered to be due to other flaviviruses.

### Entomological methods

The detailed entomological method has been published in a previous paper [23]. Briefly, selected houses were prospected to record all natural and artificial water containers (defined as ‘potential vector containers’), and immature stages (larvae and pupae) of *Ae. aegypti* and/or *Ae. albopictus*. When present, immature stages were collected for counting and identification. Larvae and pupae were returned to insectaries to count L3 and L4 larvae and pupae of *Aedes* (*Stegomyia*) species, which were then isolated from other *Culicinae* species and reared to adult stage for taxonomic identification.

### Statistical methods

Because of the contrasting city socio-environment, separated analyses were performed for each city. A univariate logistic regression with normal random effects (LRRE) was used to identify risk factors for IgG anti-DENV seropositivity controlling for the cluster effect. Odds ratios (ORs) and their 95% confidence intervals (95% CIs) were calculated. All non-collinear variables found associated (using *P*<0.25) with seropositivity in univariate analyses, were then used in a multivariate LRRE model. From the initial model including all the potential predictors, a manual backward procedure based on the likelihood-ratio test was used to remove step by step variables that were not significantly associated with the dependent variable. The Akaike Information Criteria (AIC) was used to compare the fit of non-nested models. Data analysis was performed using Stata Statistical Software release 10 (StataCorp. 2007. StataCorp LP, College Station, TX).

### Ethical aspects

The study had been approved by the National Ethics Committee of Cameroon and had received the clearance of the Ministry of Health of Cameroon (letter D30-470L/MSP/SG/DROS/CRC). Inclusion in the study was voluntary. Written informed consent was obtained from adult participants or from parents or guardians for children.

## Results

The study has been carried out in September 2006 in Garoua, in November 2006 in Douala and in December 2007 in Yaounde. Serological results were available for 2030 individuals: 728 from Garoua, 699 from Douala and 603 from Yaounde. The main characteristics of participating individuals are summarized in Table 1 (due to some missing data, the number of observations varied slightly according to variables).

**Table 1:** Main characteristics of individuals participating in the sero-epidemiologic survey of dengue in Cameroon in 2006–2007.

	Town					
Variable	Garoua		Douala		Yaounde	
	n	*%*	n	*%*	n	*%*
**Age (years)**						
2–9	64	*(8.9)*	87	*(12.5)*	89	*(13.9)*
10–19	189	*(26.2)*	110	*(15.7)*	125	*(19.5)*
20–29	155	*(21.5)*	199	*(28.5)*	210	*(32.8)*
30–44	171	*(23.7)*	188	*(26.9)*	123	*(19.2)*
≥45	143	*(19.8)*	115	*(16.5)*	94	*(14.7)*
**Sex**						
Male	344	*(49.2)*	302	*(44.7)*	255	*(39.8)*
Female	355	*(50.8)*	373	*(55.3)*	385	*(60.2)*
**DENV IgM antibodies**						
No	727	*(99.9)*	697	*(99.7)*	607	*(100.0)*
Yes	1	*(0.1)*	2	*(0.3)*	0	*(0.0)*
**DENV IgG antibodies**						
No	552	*(75.8)*	270	*(38.6)*	544	*(90.2)*
Yes	176	*(24.2)*	429	*(61.4)*	59	*(9.8)*
**IgG specificity**						
Number tested	*94*		*100*		*66*	
DENV-1	11	*(11.7)*	17	*(17.0)*	9	*(13.6)*
DENV-2	68	*(72.3)*	61	*(61.0)*	44	*(66.7)*
DENV-1+DENV-2	7	*(7.4)*	11	*(11.0)*	7	*(10.6)*
Probable other flavivirus	8	*(8.5)*	11	*(11.0)*	6	*(9.1)*

### Garoua

The median age of participating individuals was 25 years (minimum 2 years, maximum 87 years) and the males to females ratio was 0.97∶1.

Of 728 tested individuals, one was positive for anti-DENV IgM (0.1%) and 176 tested positive for anti-DENV IgG (24.2%). The individual found positive for IgM was negative for IgG, consistent with acute infection.

**Figure 2:**
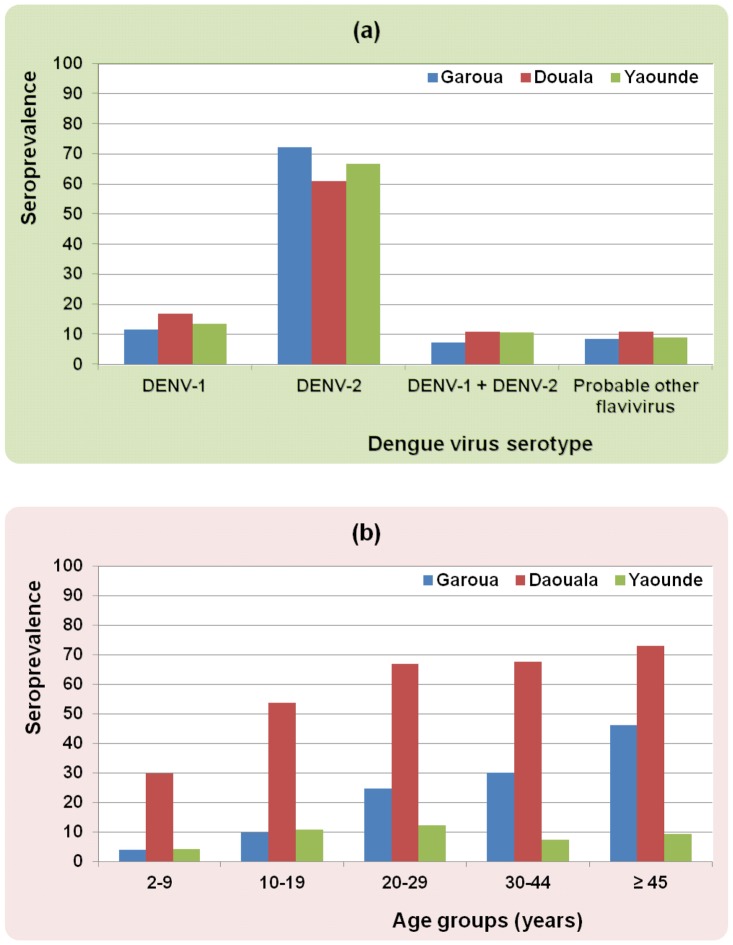
Dengue seroprevalence according to (a) serotypes and (b) age groups in the three main cities in Cameroon in 2006–2007.

Among 94 randomly identified IgG-positive sera, seroneutralization results suggest that 68 (72.3%), 11 (11.7%) and seven (7.4%) anti-DENV IgG positivities were specific of DENV-2, specific of DENV-1 and specific of both DENV-1 and DENV-2, respectively, while eight (8.5%) anti-DENV IgG positivities were not found to be specific of DENV (Table 1, Figure 2a).

The entomologic survey conducted in Garoua only observed *Ae. aegypti* larval stages. A binary variable coding for absence or presence of *Ae. aegypti* larval stages in the peridomestic area of participants was used to evaluate the association of DENV seropositivity with the exposure to vectors. In univariate analysis, age, not being born in Garoua, history of travels outside Garoua, presence of uncovered water containers in the yard of the house, absence of possession of TV at home and of cooker, showed statistically significant positive association with DENV IgG seropositivity. DENV seropositivity was neither significantly associated with reported recent history of fever, of clinical malaria, of influenza or dengue fever or to history of yellow fever immunization, nor was associated with the presence of larval stages of *Ae. aegypti*. Twenty variables (listed in Table S1) were considered eligible for multivariate analysis.

From the initial multivariate model including 20 variables, four variables remained independently associated with DENV seropositivity with p-values<0.05 and a fifth had a p-value = 0.053. Despite this p-value slightly over 0.05, the variable, “breeding pigs in the yard”, has been kept since its removal would have led to a significant change of the likelihood. Therefore, we retained the 5-variable model comprising the following predictors: age, presence of uncovered water containers in the yard of the house, no possession of TV at home, not being born in Garoua and absence of breeding pigs in the yard (Table 2). The likelihood ratio test for the cluster effect was not significant (*p = 0.41*).

**Table 2:** Sero-epidemiologic survey of dengue in Garoua, Cameroon in 2006–2007: Multivariate analysis using logistic regression with random effect.

Risk factor	No. tested	% IgG	Adjusted OR	OR 95%CI	p
**Age group (years)**					
2–9	61	*3.3*	1		
10–19	185	*9.7*	3.1	(0.7–13.9)	0.14
20–29	153	*24.8*	9.2	(2.1–40.1)	<0.01
30–44	170	*30.0*	10.2	(2.4–44.1)	<0.01
≥45	141	*46.1*	18.1	(4.2–79.1)	<0.01
**Being born outside Garoua**					
No	343	*14.0*	1		
Yes	367	*34.3*	2.2	(1.4–3.3)	<0.01
**Having a TV at home**					
Yes	388	*19.6*	1		
No	322	*30.4*	1.6	(1.1–2.4)	0.01
**Uncovered water container**					
No	638	*23.4*	1		
Yes	72	*34.7*	2.0	(1.1–3.6)	0.02
**Breeding pigs in the yard**					
Yes	23	*8.7*	1		
No	687	*25.0*	4.5	(1.0–20.9)	0.05

### Douala

Females represented 55.3% of our sample, compared to 50% expected. The median age of all participating individuals was 28 years (minimum 2 years, maximum 99 years).

Overall, 2 individuals tested positive for anti-DENV IgM (0.3%) and 429 tested positive for anti-DENV IgG (61.4%). The two sera tested positive for IgM were also tested positive for IgG. Using seroneutralization test on 100 randomly identified IgG-positive sera, 61 (61%) had antibodies against DENV-2, 17 (17%) had antibodies specific of DENV-1 and 11 (11%) had antibodies against both DENV-1 and DENV-2, while 11 (11%) had IgG antibodies not specific for DENV (Table 1, Figure 2a). In univariate analysis, age and a history of travel outside of Douala were significantly associated with DENV seropositivity. Neither present illness nor presence of symptoms evocative of dengue fever, such as fever, headache, myalgia, arthralgia or rash were associated with DENV seropositivity. DENV seropositivity was not associated with history of yellow fever immunization or to reported recent history of malaria, of influenza or dengue fever. Only one person (0.1%) reported having presented suspected dengue fever during the last three months, while 183 reported having suffered from clinical malaria (25.6%). Neither the use of bednet nor the use of repellent was associated with DENV seropositivity. Some housing or environmental characteristics were significantly associated with DENV seropositivity: house walls made of temporary materials, house walls not covered, private toilets located outside, old tires lying around in the yard, water storage in the yard, uncovered water containers, domestic waste water out in the open. Of different variables accounting for the presence or number of larval stages of *Ae. aegypti* and/or *Ae. albopictus* collected in the environment of investigated houses, only the presence/absence of larval stages of *Ae. aegypti* and the abundance of larval stages of *Ae. aegypti* coding for 3 modalities had p-values<0.25 although >0.05. The main results of univariate analysis are summarized in Table S2.

In multivariate analysis, the initial model included 22 variables. The variable “History of travels outside Douala”, led to a significant change in the likelihood. This variable was then kept in the final multivariate model, although the p-value was slightly over 0.05 (0.055). Therefore, we retained the 8-variable model comprising the following predictors: age-group, house walls materials, water-storage containers in the yard, old tires in the yard, location of toilets, having no TV at home, having no air conditioning and history of travels outside Douala (Table 3).

**Table 3:** Sero-epidemiologic survey of dengue in Douala, Cameroon in 2006–2007: Multivariate analysis using logistic regression with random effect.

Risk factor	No. tested	% IgG	Adjusted OR	OR 95%CI	p
**Age group (years)**					
2–9	85	*29.4*	1		
10–19	104	*54.8*	3.9	(1.9–8.0)	<0.01
20–29	190	*67.4*	7.6	(3.9–14.9)	<0.01
30–44	180	*67.2*	8.7	(4.3–17.5)	<0.01
≥45	107	*73.8*	13.1	(5.9–29.1)	<0.01
**History of travels outside Douala**					
Never	59	*42.4*	1		
At least once	607	*63.4*	2	(1.0–4.0)	0.06
**Home ventilation**					
Air conditioning	12	*16.7*	1		
Fan	493	*61.5*	10.5	(1.8–61.6)	0.01
Natural	161	*65.2*	9.1	(1.5–56.8)	0.02
**Having TV at home**					
Yes	518	*58.1*	1		
No	148	*73.6*	2	(1.2–3.4)	0.01
**House walls materials**					
Not temporary	442	*57.5*	1		
Temporary	224	*69.6*	2.3	(1.4–3.8)	<0.01
**Location of toilets**					
Inside home	199	*56.8*	1		
Outside, private	290	*69.7*	2.2	(1.3–3.8)	<0.01
Outside, shared	177	*53.7*	1.6	(0.9–2.8)	0.10
**Old tires in the yard**					
0	475	*58.5*	1		
≥1	191	*69.1*	1.7	(1.1–2.7)	0.02
**Water storage in the yard**					
No	493	*57.2*	1		
Yes	173	*74.0*	1.9	(1.1–3.3)	0.02

The likelihood ratio test for the presence of a cluster effect was significant (*p*<10^−3^), indicating a correlation between IgG status among individuals belonging to the same cluster, after controlling on other variables.

### Yaounde

The male-to-female sex ratio fell as low as 0.66 in this city.

The overall proportion of IgG positive individuals was 9.8%, without any significant difference between age groups (Figure 2b) or even any sign evocative of a possible trend. None of the tested individuals was positive for DENV IgM. The seroneutralization test performed on 66 randomly identified IgG-positive sera showed that 44 (66.7%) had antibodies against DENV-2, nine (13.6%) had antibodies specific of DENV-1 and seven (10.6%) had antibodies against both DENV-1 and DENV-2, while six (8.0%) had IgG antibodies not specific for DENV (Table 1, Figure 2a).

Of the 59 IgG-positive individuals, 93.2% had already travelled outside Yaounde and 74% had been in the region of Douala, not to mention other destinations.

The results of univariate analysis are presented in Table S3.

Our initial multivariate model for IgG seropositivity included 11 variables, of which three were kept in the final model: reporting having had a malaria access within the last three months (OR 2.3; 95% CI: 1.3–4.1), having banana trees in the yard of the house (OR 2.6; 95% CI: 1.4–4.8) and presence of stagnant water in the yard at the time of the survey (OR 1.9; 95% CI: 1.1–3.5) (Table 4). The likelihood ratio test for the presence of a cluster effect was not significant (*p = 0.49*).

**Table 4:** Sero-epidemiologic survey of dengue in Yaounde, Cameroon in 2006–2007: Multivariate analysis using logistic regression with random effect.

Risk factor	No. tested	% IgG	Adjusted OR	OR 95%CI	p
**Malaria access within the last 3 months**					
No	422	*7.6*	1		
Yes	154	*14.3*	2.3	(1.3–4.1)	0.01
**Stagnant water in the yard**					
No	421	*8.1*	1		
Yes	155	*12.9*	1.9	(1.1–3.5)	0.03
**Banana trees in the yard**					
No	430	*7.4*	1		
Yes	146	*15.1*	2.6	(1.4–4.8)	<0.01

## Discussion

The epidemiology of dengue fever was still poorly documented in Cameroon, and the present study is the first aiming at assessing the frequency of human infection and at documenting risk factors of DENV infection in this country.

The first finding of this study was the heterogeneity of prevalence of anti-DENV IgG between surveyed towns (Figure 2a). In fact, this observation was consistent with the heterogeneity of entomological parameters as reported by Kamgang *et al.* [23]. *Aedes aegypti* was the most abundant species in Douala, as was *Ae. albopictus* in Yaounde, while *Ae. aegypti* was the only species found in Garoua.

DENV-1 and DENV-2 have been, or are currently, circulating in Cameroon. These results are consistent with those of Kuniholm *et al.* [13] in rural areas of South Cameroon but contrast with absence of DENV infections among several cases of dengue-like illness (diagnosed as Chikungunya cases) in three villages of West region in 2007 [25]. Nevertheless, there is no evidence on the epidemiological pattern since dengue fever is not monitored and there is no report of past epidemics. On the contrary, following an active surveillance of arboviruses in Gabon between 2007 and 2010, concomitant outbreaks of DENV and Chikungunya virus were recorded [10], [11]. Absence of reported DENV epidemic in Cameroon may be due to the fact that the vast majority of infections by DENV are unapparent or oligosymptomatic and that this form of presentation of the disease may be more common than in other areas [26].

It has been demonstrated that nonneutralizing antibodies raised by natural infection with one of the four DENV may enhance infection with a different virus by “intrinsic antibody-dependant enhancement” process [27]. Despite the circulation of two serotypes (DENV-1 and 2) and the fact that 7 to 11% of the study population were infected by both DENV-1 and 2, no Dengue Hemorrhagic Fever (DHF) case has been reported in the country. The lack of records of occurrence of DHF in Cameroon and in Gabon [11] may be due to the confusion of DHF with clinical malaria by non-specialists medical personnel among other factors [28]. Discussion on the low incidence of DHF in Africa has been quite intense in the literature and African ancestry appeared to be relatively protective for severe forms of dengue but not for classic dengue fever [21], [29]. Although 61% of the study population in Douala were tested positive for anti-DENV IgG, few people have heard about dengue fever. It is remarkable that only one individual reported having suffered from dengue fever during the last three months before interview, while 183 reported having presented a clinical malaria attack. However, fever episodes are most often considered as malaria without any laboratory confirmation, and in fact, some of the supposed malaria attacks could be actual dengue fever. The latter remains largely unknown and its biological diagnosis is exceptionally ordered by physicians. The same ignorance of dengue was observed in Garoua and Yaounde, with respectively 1.1% and 3.7% of participating individuals having heard about dengue.

Age is the individual variable the most constantly associated with DENV seroprevalence [30]–[33]. The very low proportion of individuals with anti-DENV IgM suggests that DENV transmission is not permanent in Douala, in Garoua and in Yaounde. Since anti-DENV IgG may persist for several decades [34], [35], the increasing proportion of anti-DENV IgG positivity with increasing age (Figure 2b) indicates either that several epidemics have occurred in Douala and in Garoua in past years at regular intervals, or that there is a relatively stable endemic transmission of DENV as demonstrated in Gabon by Caron *et al.* [11]. These data show that the epidemiology of dengue, with regard to the characteristics of people is similar to that observed for a long period in Brazil and in many other countries in the Americas [36]–[38], especially in relation to the increase of the seropositivity as the age increases, reaching predominantly in adults. This age distribution of the disease is different in many countries of Asia although recent studies revealed age shift which might be a consequence of the demographic transitions [39]. In Yaounde, the relatively constant, low proportion of IgG-positive individuals according to age groups (Figure 2b) could suggest a single recent outbreak, or, alternatively, DENV infections contracted outside Yaounde.

Males and young age groups were under-represented in our samples from Douala and from Yaounde, as it is often the case in surveys in urban settings, where most men have to leave home for work and young people are attending school. In our survey, the gender was not associated with DENV IgG seropositivity. Some studies have reported a higher risk of DENV IgG seroprevalence in males than in females [22], [31], but elsewhere females were found having a higher risk than males [23], and no difference was observed between genders in other settings [32], [40]. It is likely that specific, not identified, occupational or behavioural risk factors account for these contradictory findings from different areas.

In our multivariate model for Douala, five out of eight independent risk factors for DENV IgG seropositivity were related to housing quality: house walls made of temporary materials, private toilets located outside the house, absence of air-conditioning, domestic water stored in the yard, presence of old tires in the yard. None of these factors was significant in Garoua, or in Yaounde. Having toilets located outside the house had been previously identified as a risk factor of DENV infection in a survey in Puerto Rico [18]. However, we observed that in Douala the risk was associated with private toilets but not to toilets being shared by several families and we could not find any satisfactory explanation for that.

Like in our survey in Douala, air-conditioning, which is usually associated with closed windows, had been found protecting against DENV infections in Texas [41]. It is nevertheless unlikely that the very low proportion of equipped houses can significantly increase in the near future because of the cost of air conditioners and electricity.

The significant risk associated with used automobile tires discarded around houses in Douala is consistent with the knowledge that tires are a good larval habitat for *Ae. aegypti* and *Ae. albopictus* [19]. A survey in southern Cameroon has shown that used tires, discarded tin cans, plastic containers of all sorts, earthenware jars, abandoned car parts and brick holes were the most common larval habitat for both *Ae. albopictus* and *Ae. aegypti* [15]. However, apart from tires, we have not observed any significant relationship between the presence of other rubbish littered in the yards and DENV seropositivity. As in this study, the use of containers to store domestic water, a consequence of the lack of piped water, has been sometimes associated with higher risk of DENV infection [42], although some authors consider that small disposable receptacles are predominant breeding sites for the vectors [40].

Our questionnaire did not include direct assessment of monetary income, because reliability of answers to that kind of question is often questionable [43]. However, not having a TV at home, which was associated with an increased risk of anti-DENV IgG seropositivity, must be considered as a proxy for household income, independently of poor housing conditions. This marker of poverty could have accounted for other risk factors not studied in our survey, such as having a rubbish heap, or scrub or other close to home.

Some individual or household factors were not found to be independently associated with DENV seropositivity in our survey, while sometimes identified in the univariate analysis or in other surveys (e.g. educational level [40], window screens [18], presence of dogs or cats [32]).

The absence of variables accounting for presence, or abundance, of larval stages of vector mosquitoes in our multivariate model is not surprising. The entomological survey was carried out in a single visit. Although very useful to document the presence of *Aedes* vectors, the snapshot taken during the survey was not enough to draw a reliable picture of the variability of vector abundance over the year. Even when carried out together with dengue disease incidence, entomological surveys may fail to evidence a relationship between household mosquito counts and recent dengue infection [44].

Despite the borderline association in multivariate analysis between DENV seropositivity and history of travel outside Douala, we could not further delve into this information because of the large number of reported destinations, of which a great proportion could not be located precisely.

The borderline association, observed in Garoua, between anti-DENV IgG seronegativity and swine breeding next to the house is counterintuitive. Since *Ae. aegypti*, as well as *Ae. albopictus* are known to prefer humans hosts for blood feeding [45], the presence of pigs should not protect humans from being bitten, but one cannot reject the hypothesis that the presence of pigs could lead to a kind of dilution of mosquito bites resulting in a lower risk of DENV transmission to humans. The observed relationship may also be due to chance or a confounding factor, unless during their search for food, pigs contribute to wipe out potential breeding sites.

In Douala, the significant random effect associated with clusters can be better understood considering the focal nature of dengue with some risk factors varying over time [46]. Although no significant random effect was observed in Garoua and Yaounde, we believe that using LRRE was fully justified, in view of the sampling scheme.

Our study had several limitations. First, seroneutralization performed on a random subsample of 260 sera showed that, overall, 9.6% of IgG positive individuals had antibodies not specific for DENV. If up to one-tenth of ELISA-positive individuals may have been wrongfully considered as having DENV antibodies, we cannot rule out a possible impact on the risk factors analysis. Another limitation is that we cannot ensure that the observations on housing and environment that were made during the survey faithfully represent the conditions that prevailed at the time when DENV seropositive persons have been infected, possibly many years ago. Another potential limitation is that the survey did not explore the characteristics of places where people can stay several hours every day for working or teaching. Adams and Kapan have suggested that some places that people visit frequently and briefly can play a role of hubs and reservoirs of transmission [47]. Ordinary movements of the population would deserve detailed investigation in order to better document the main places where DENV transmission can occur and if some occupational characteristics may be considered as risk factors [48]. As well, other possible limit is that some risk factor present in a given residence may have affected people living very close to this residence, although this factor is missing in their home (eg used tires, rubbish, absence of domestic waste water drainage, washing place in the yard, etc.), with the possible effect of hiding or lessening the role of this factor. The positive random effect could be an evidence of this. Lastly, although the persistence of DENV IgG is long, one cannot rule out a possible inter-individual variability in persistence leading to underestimation of the proportion of infected individuals.

DENV transmission risk factors are essentially linked to underdevelopment. The ways to reduce the transmission are long term development and improvement of sanitation and education. Information campaign on this disease and the way to avoid it should be developed by local populations and national authorities.

Our study has shown that DENV infection is not uncommon in Cameroon, although unrecognized by individuals and even by medical personnel. There is a need for assessing the disease burden of dengue fever by encouraging health professionals to think about dengue fever and to prescribe laboratory diagnosis of dengue when appropriate. The integration of dengue fever within the epidemiological surveillance in Cameroon would be an invaluable means of documenting the epidemiological pattern of dengue fever in Cameroon and fight this disease.

## Supporting Information

Checklist S1 **STROBE Checklist for cross-sectional studies.**
(DOC)

Table S1 **Sero-epidemiologic survey of dengue in Garoua Cameroon in 2006–2007: Univariate analysis using logistic regression with random effect.**
(DOC)

Table S2 **Sero-epidemiologic survey of dengue in Douala, Cameroon in 2006–2007: Univariate analysis using logistic regression with random effect.**
(DOC)

Table S3 **Sero-epidemiologic survey of dengue in Yaounde, Cameroon in 2006–2007: Univariate analysis using logistic regression with random effect.**
(DOC)
