# Supplementary material for: Evidence of Dengue Virus Transmission and Factors Associated with the Presence of Anti-Dengue Virus Antibodies in Humans in Three Major Towns in Cameroon
Source: PLoS Negl Trop Dis. 2014 Jul 10;8(7):e2950. doi: 10.1371/journal.pntd.0002950 (PMC4091864; doi:10.1371/journal.pntd.0002950)
Supplement: Checklist S1 — STROBE Checklist for cross-sectional studies. (DOC) [file pntd.0002950.s001.doc]

STROBE Statement—Checklist of items that should be included in reports of ***cross-sectional studies***

|  | Item No | Recommendation |
| --- | --- | --- |
| **Title and abstract** | 1 | 1. Indicate the study’s design with a commonly used term in the title or the abstract   The study is a cross sectional survey using a random cluster sampling design. |
|  |  | 1. Provide in the abstract an informative and balanced summary of what was done and what was found   On the basis of anti-dengue virus IgG seropositivity in humans, our survey demonstrated that dengue virus transmission occurred in the three main towns of the country. Although the findings varied according to the location, identified risk factors of anti-dengue virus seropositivity were commonly related to housing conditions. |
| Introduction | | |
| Background/rationale | 2 | Explain the scientific background and rationale for the investigation being reported  Dengue is not well documented in Africa. In Cameroon, data are scarce, but dengue infection has been confirmed in humans. |
| Objectives | 3 | State specific objectives, including any prespecified hypotheses  This study was to document risk factors associated with anti-dengue virus Immunoglobulin G seropositivity in humans in three major towns in Cameroon. |
| Methods | | |
| Study design | 4 | Present key elements of study design early in the paper  The study is a cross sectional survey carried out in each town, using a random cluster sampling method based on cluster identification from geographical coordinates. |
| Setting | 5 | Describe the setting, locations, and relevant dates, including periods of recruitment, exposure, follow-up, and data collection  The study has been carried out in September 2006 in Garoua, in November 2006 in Douala and in December 2007 in Yaounde. |
| Participants | 6 | 1. Give the eligibility criteria, and the sources and methods of selection of participants   All individuals >9 months were invited to participate. All participating individuals were interviewed using a standardized questionnaire form. |
| Variables | 7 | Clearly define all outcomes, exposures, predictors, potential confounders, and effect modifiers. Give diagnostic criteria, if applicable  Outcome: anti-dengue virus IgG seropositivity. Predictors: more than 25 variables were recorded with a tenth of predictors (age, presence of uncovered water containers in the yard of the house, no possession of TV at home, not being born in town, absence of breeding pigs in the yard, house walls materials, water-storage containers in the yard, old tires in the yard, location of toilets, having no air conditioning and history of travels outside the town) |
| Data sources/ measurement | 8* | For each variable of interest, give sources of data and details of methods of assessment (measurement). Describe comparability of assessment methods if there is more than one group  Information that was common to all individuals belonging to the same household was recorded from the head of the family. Standardized environmental and housing characteristics were collected by the investigators.  A blood sample was collected for all volunteers during household visit in each town.  Selected houses were prospected to record all natural and artificial water containers and immature stages of *Ae. aegypti* and/or *Ae. albopictus*. |
| Bias | 9 | Describe any efforts to address potential sources of bias  We cannot ensure that the observations on housing and environment that were made during the survey faithfully represent the conditions that prevailed at the time when DENV seropositive persons have been infected, possibly many years ago. Another potential limitation is that the survey did not explore the characteristics of places where people can stay several hours every day for working or teaching. |
| Study size | 10 | Explain how the study size was arrived at  The sampling plan consisted in randomization of 75 GPS (Global Positioning System) points within each town, with probability proportional to the population density of districts according to the 3^rd^ General Population and Housing Census of Cameroon (3^rd^ GPHC, 2005). |
| Quantitative variables | 11 | Explain how quantitative variables were handled in the analyses. If applicable, describe which groupings were chosen and why  No Groupings were chosen for variables. |
| Statistical methods | 12 | 1. Describe all statistical methods, including those used to control for confounding   Because of the contrasting city socio-environment, separated analyses were performed for each city. A univariate logistic regression with normal random effects (LRRE) was used to identify risk factors for IgG anti-DENV seropositivity controlling for the cluster effect.  All non-collinear variables found associated (using *P* < 0.25) with seropositivity in univariate analyses, were then used in a multivariate LRRE model. From the initial model including all the potential predictors, a manual backward procedure based on the likelihood-ratio test was used to remove step by step variables that were not significantly associated with the dependent variable.  The Akaike Information Criteria (AIC) was used to compare the fit of non-nested models. |
|  |  | (*b*) Describe any methods used to examine subgroups and interactions  No interactions were tested. |
|  |  | (*c*) Explain how missing data were addressed  Missing data were considered to have occurred at random. Individual and household with missing data were discarded. The number of observations varied slightly according to variables. |
|  |  | (d)If applicable, describe analytical methods taking account of sampling strategy  Univariate and multivariate logistic regression with normal random effects (LRRE) was used to identify risk factors for IgG anti-DENV seropositivity controlling for the cluster effect. |
|  |  | 1. Describe any sensitivity analyses   Not applicable |
| Results | | |
| Participants | 13* | 1. Report numbers of individuals at each stage of study—eg numbers potentially eligible, examined for eligibility, confirmed eligible, included in the study, completing follow-up, and analysed   The study has been carried out in September 2006 in Garoua, in November 2006 in Douala and in December 2007 in Yaounde.  790, 730 and 700 participants were sampled respectively in Garoua, Douala and Yaounde. Serological results were available for 2030 individuals: 728 from Garoua, 699 from Douala and 603 from Yaounde. |
|  |  | (b) Give reasons for non-participation at each stage  Only few refusals were observed |
|  |  | (c) Consider use of a flow diagram  None considered |
| Descriptive data | 14* | 1. Give characteristics of study participants (eg demographic, clinical, social) and information on exposures and potential confounders   See table |
|  |  | 1. Indicate number of participants with missing data for each variable of interest   62, 31 and 97 samples respectively from Garoua, Douala and Yaounde were not tested because of the insufficient quantity or poor quality of sera. |
| Outcome data | 15* | Report numbers of outcome events or summary measures  Anti-dengue IgG were found from 61.4% of sera in Douala (n=699), 24.2% in Garoua (n=728) and 9.8% in Yaounde (n=603). IgM were found from 0.3% of Douala samples, 0.1% of Garoua samples and 0.0% of Yaounde samples. Seroneutralization on randomly selected IgG positive sera showed that 72% (n = 100) in Douala, 80% (n = 94) in Garoua and 77% (n = 66) in Yaounde had antibodies specific for dengue virus serotype 2 (DENV-2). |
| Main results | 16 | 1. Give unadjusted estimates and, if applicable, confounder-adjusted estimates and their precision (eg, 95% confidence interval). Make clear which confounders were adjusted for and why they were included   See table |
|  |  | 1. Report category boundaries when continuous variables were categorized   Non applicable |
|  |  | 1. If relevant, consider translating estimates of relative risk into absolute risk for a meaningful time period   Non applicable |
| Other analyses | 17 | Report other analyses done—eg analyses of subgroups and interactions, and sensitivity analyses  Not applicable |
| Discussion | | |
| Key results | 18 | Summarise key results with reference to study objectives  Age, temporary house walls materials, having water-storage containers, old tires or toilets in the yard, having no TV, having no air conditioning and having travelled at least once outside the city were independently associated with anti-dengue IgG positivity in Douala. Age, having uncovered water containers, having no TV, not being born in Garoua and not breeding pigs were significant risk factors in Garoua. Recent history of malaria, having banana trees and stagnant water in the yard were independent risk factors in Yaounde. |
| Limitations | 19 | Discuss limitations of the study, taking into account sources of potential bias or imprecision. Discuss both direction and magnitude of any potential bias  Our study had several limitations. First, seroneutralization performed on a random subsample of 260 sera showed that, overall, 9.6% of IgG positive individuals had antibodies not specific for DENV. If up to one-tenth of ELISA-positive individuals may have been wrongfully considered as having DENV antibodies, we cannot rule out a possible impact on the risk factors analysis. Another limitation is that we cannot ensure that the observations on housing and environment that were made during the survey faithfully represent the conditions that prevailed at the time when DENV seropositive persons have been infected, possibly many years ago. Another potential limitation is that the survey did not explore the characteristics of places where people can stay several hours every day for working or teaching. Adams and Kapan have suggested that some places that people visit frequently and briefly can play a role of hubs and reservoirs of transmission. Ordinary movements of the population would deserve detailed investigation in order to better document the main places where DENV transmission can occur and if some occupational characteristics may be considered as risk factors. As well, other possible limit is that some risk factor present in a given residence may have affected people living very close to this residence, although this factor is missing in their home (eg used tires, rubbish, absence of domestic waste water drainage, washing place in the yard, etc.), with the possible effect of hiding or lessening the role of this factor. The positive random effect could be an evidence of this. Lastly, although the persistence of DENV IgG is long, one cannot rule out a possible inter-individual variability in persistence leading to underestimation of the proportion of infected individuals. |
| Interpretation | 20 | Give a cautious overall interpretation of results considering objectives, limitations, multiplicity of analyses, results from similar studies, and other relevant evidence  The significant random effect associated with clusters observed in Douala can be better understood considering the focal nature of dengue with some risk factors varying over time. Although no significant random effect was observed in Garoua and Yaounde, we believe that using LRRE was fully justified, in view of the sampling scheme. |
| Generalisability | 21 | Discuss the generalisability (external validity) of the study results  DENV transmission risk factors are essentially linked to underdevelopment. |
| Other information | | |
| Funding | 22 | Give the source of funding and the role of the funders for the present study and, if applicable, for the original study on which the present article is based  This study was supported by the French government (Agence Nationale pour la Recherche) through the Epidengue project (ANR 05 SEST 010 -01). The funders had no role in study design, data collection and analysis, decision to publish, or preparation of the manuscript. |

*Give information separately for exposed and unexposed groups.

**Note:** An Explanation and Elaboration article discusses each checklist item and gives methodological background and published examples of transparent reporting. The STROBE checklist is best used in conjunction with this article (freely available on the Web sites of PLoS Medicine at http://www.plosmedicine.org/, Annals of Internal Medicine at http://www.annals.org/, and Epidemiology at http://www.epidem.com/). Information on the STROBE Initiative is available at www.strobe-statement.org.
