# Supplementary material for: Evidence of Dengue Virus Transmission and Factors Associated with the Presence of Anti-Dengue Virus Antibodies in Humans in Three Major Towns in Cameroon
Source: PLoS Negl Trop Dis. 2014 Jul 10;8(7):e2950. doi: 10.1371/journal.pntd.0002950 (PMC4091864; doi:10.1371/journal.pntd.0002950)
Supplement: Table S1 — Sero-epidemiologic survey of dengue in Garoua Cameroon in 2006–2007: Univariate analysis using logistic regression with random effect. (DOC) [file pntd.0002950.s002.doc]

| **Risk factor** | **No. tested** | **% IgG** | **Univariate OR** | **OR 95%CI** | **p** |
| --- | --- | --- | --- | --- | --- |
| **Age group (years)** | | | | | |
| 2-9 | 61 | 3.9 | 1 |  |  |
| 10-19 | 185 | 9.7 | 3.1 | (0.7-14.2) | 0.13 |
| 20-29 | 153 | 24.8 | 9.7 | (2.3-41.7) | <0.01 |
| 30-44 | 170 | 30.0 | 12.7 | (3.0-54.1) | <0.01 |
| ≥ 45 | 141 | 46.1 | 25.5 | (6.0-108.8) | <0.01 |
| **Being born outside Garoua** | | | | | |
| No | 352 | 14.2 | 1 |  |  |
| Yes | 376 | 33.5 | 3.2 | (2.2-4.8) | <0.01 |
| **History of travels outside Garoua** | | | | | |
| Never | 185 | 14.1 | 1 |  |  |
| At least once | 543 | 27.6 | 2.3 | (1.5-3.7) | <0.01 |
| **Fever episode within the last 3 months** | | | | | |
| No | 522 | 22.8 | 1 |  |  |
| Yes | 226 | 27.7 | 1.3 | (0.9-1.9) | 0.19 |
| **Malaria access within the last 3 months** | | | | | |
| No | 576 | 22.7 | 1 |  |  |
| Yes | 152 | 29.6 | 1.4 | (0.9-2.2) | 0.09 |
| **Home ventilation** | | | | | |
| Air conditioning | 26 | 11.5 | 1 |  |  |
| Fan | 350 | 22.6 | 2.4 | (0.7-8.7) | 0.17 |
| Natural | 348 | 27.0 | 3.1 | (0.9-11.1) | 0.08 |
| **Having a TV at home** | | | | | |
| Yes | 392 | 19.4 | 1 |  |  |
| No | 336 | 29.8 | 1.8 | (1.2-2.5) | <0.01 |
| **Having a cooker at home** | | | | | |
| Yes | 74 | 13.5 | 1 |  |  |
| No | 654 | 25.4 | 2.2 | (1.1-4.5) | 0.03 |
| **House walls covering** | | | | | |
| Yes | 643 | 23.0 | 1 |  |  |
| No | 82 | 30.5 | 1.4 | (0.8-2.4) | 0.18 |
| **Cemented bathroom walls** | | | | | |
| Yes | 261 | 20.7 | 1 |  |  |
| No | 451 | 26.2 | 1.3 | (0.9-2.0) | 0.12 |
| **Location of toilets** | | | | | |
| Inside home | 71 | 14.1 | 1 |  |  |
| Outside, private | 351 | 23.9 | 1.9 | (0.9-4.0) | 0.09 |
| Outside, shared | 282 | 27.7 | 2.3 | (1.1-4.9) | 0.03 |
| **Uncovered water containers** | | | | | |
| Not any | 656 | 23.0 | 1 |  |  |
| ≥ 1 | 72 | 34.7 | 1.8 | (1.0-3.1) | 0.04 |
| **Type of ground** | | | | | |
| Hillside | 349 | 20.9 | 1 |  |  |
| Gully | 74 | 23.0 | 1.1 | (0.6-2.1) | 0.74 |
| Flat ground | 305 | 28.2 | 1.5 | (1.0-2.2) | 0.04 |
| **Fenced yard** | | | | | |
| Yes, fully | 448 | 24.8 | 1 |  |  |
| Yes, partly | 231 | 21.7 | 0.7 | (0.5-1.2) | 0.18 |
| No | 45 | 31.1 | 1.4 | (0.7-2.9) | 0.33 |
| **Rubbish littered in the yard** | | | | | |
| Yes | 404 | 21.8 | 1 |  |  |
| No | 298 | 27.9 | 1.4 | (1.0-2.1) | 0.05 |
| **Water tank in the yard** | | | | | |
| No | 501 | 22.8 | 1 |  |  |
| Yes | 214 | 28.5 | 1.4 | (0.9-2.0) | 0.11 |
| **Stagnant water in the yard** | | | | | |
| No | 545 | 23.1 | 1 |  |  |
| Yes | 137 | 29.2 | 1.4 | (0.9-2.2) | 0.11 |
| **Coconut trees in the yard** | | | | | |
| 0 | 720 | 23.9 | 1 |  |  |
| ≥ 1 | 7 | 42.9 | 2.7 | (0.5-13.9) | 0.22 |
| **Breeding pigs in the yard** | | | | | |
| Yes | 23 | 8.7 | 1 |  |  |
| No | 705 | 24.7 | 4.5 | (1.0-21.3) | 0.06 |
| **L3 + L4 + pupae of *Ae. aegypti*** | | | | | |
| Absence | 480 | 22.9 | 1 |  |  |
| Presence | 248 | 26.6 | 1.3 | (0.9-1.8) | 0.24 |
