# Supplementary material for: Evidence of Dengue Virus Transmission and Factors Associated with the Presence of Anti-Dengue Virus Antibodies in Humans in Three Major Towns in Cameroon
Source: PLoS Negl Trop Dis. 2014 Jul 10;8(7):e2950. doi: 10.1371/journal.pntd.0002950 (PMC4091864; doi:10.1371/journal.pntd.0002950)
Supplement: Table S2 — Sero-epidemiologic survey of dengue in Douala, Cameroon in 2006–2007: Univariate analysis using logistic regression with random effect. (DOC) [file pntd.0002950.s003.doc]

| **Risk factor** | **No. tested** | **% IgG** | **Univariate OR** | **OR 95%CI** | **p** |
| --- | --- | --- | --- | --- | --- |
| **Age group (years)** | | | | | |
| 2-9 | 87 | *29.9* | 1 |  |  |
| 10-19 | 110 | *53.6* | 3.6 | (1.8-6.9) | <0.01 |
| 20-29 | 199 | *66.8* | 6.7 | (3.6-12.5) | <0.01 |
| 30-44 | 188 | *67.6* | 7.8 | (4.1-14.9) | <0.01 |
| ≥ 45 | 115 | *73.0* | 10.1 | (4.9-20.7) | <0.01 |
| **History of travels outside Douala** | | | | | |
| Never | 64 | *43.8* | 1 |  |  |
| At least once | 632 | *63.4* | 2.3 | (1.3-4.2) | <0.01 |
| **Use of topical repellents** | | | | | |
| Often | 120 | *60.0* | 1 |  |  |
| Sometimes | 53 | *70.0* | 1.8 | (0.8-4.0) | 0.17 |
| Never | 320 | *61.0* | 0.8 | (0.5-1.3) | 0.28 |
| **Home ventilation** | | | | | |
| Air conditioning | 12 | *16.7* | 1 |  |  |
| Fan | 510 | *60.6* | 8.8 | (1.6-47.2) | 0.01 |
| Natural | 172 | *66.3* | 10.3 | (1.9-57.2) | 0.01 |
| **Having a TV at home** | | | | | |
| Yes | 545 | *58.0* | 1 |  |  |
| No | 157 | *74.1* | 2 | (1.3-3.1) | <0.01 |
| **House walls materials** | | | | | |
| Not temporary | 466 | *56.9* | 1 |  |  |
| Temporary | 230 | *70.0* | 1.8 | (1.2-2.7) | <0.01 |
| **House walls covering** | | | | | |
| Yes | 481 | *57.8* | 1 |  |  |
| No | 206 | *68.9* | 1.7 | (1.1-2.6) | 0.01 |
| **Number of floors** | | | | | |
| 1 | 649 | *62.1* | 1 |  |  |
| 2 | 39 | *58.9* | 0.9 | (0.4-1.9) | 0.76 |
| ≥ 3 | 9 | *22.2* | 0.1 | (0.0-1.0) | 0.05 |
| **Type of home floor** | | | | | |
| Cemented | 614 | *59.9* | 1 |  |  |
| Earthen | 85 | *71.8* | 1.6 | (0.9-3.0) | 0.10 |
| **Cemented bathroom walls** | | | | | |
| No | 214 | *68.7* | 1 |  |  |
| Yes | 454 | *57.9* | 0.6 | (0.4-0.9) | 0.04 |
| **Location of toilets** | | | | | |
| Inside home | 212 | *56.6* | 1 |  |  |
| Outside, private | 292 | *69.5* | 1.8 | (1.1-2.8) | 0.02 |
| Outside, shared | 178 | *53.9* | 0.8 | (0.5-1.3) | 0.38 |
| **Uncovered water containers** | | | | | |
| 0 | 530 | *57.9* | 1 |  |  |
| ≥ 1 | 169 | *72.2* | 1.6 | (1.0-2.6) | 0.04 |
| **Type of ground** | | | | | |
| Flat ground | 263 | *56.7* | 1 |  |  |
| Hillside | 287 | *63.1* | 1.3 | (0.8-1.9) | 0.29 |
| Gully | 149 | *66.4* | 1.7 | (0.9-3.0) | 0.08 |
| **Surface area of the yard** | | | | | |
| < 200 m² | 193 | *68.4* | 1 |  |  |
| 200-400 m² | 326 | *60.1* | 0.7 | (0.4-1.1) | 0.11 |
| > 400 m² | 180 | *56.1* | 0.6 | (0.4-1.0) | 0.07 |
| **Domestic waste water drainage** | | | | | |
| Closed pipes | 134 | *48.5* | 1 |  |  |
| Out in the open | 565 | *64.4* | 1.9 | (1.2-3.1) | 0.01 |
| **Rubbish littered in the yard** | | | | | |
| No | 552 | *59.4* | 1 |  |  |
| Yes | 147 | *68.7* | 1.3 | (0.8-2.1) | 0.24 |
| **Water tank in the yard** | | | | | |
| No | 572 | *59.1* | 1 |  |  |
| Yes | 127 | *71.7* | 1.5 | (0.9-2.6) | 0.09 |
| **Old tires in the yard** | | | | | |
| 0 | 492 | *58.3* | 1 |  |  |
| ≥ 1 | 202 | *69.3* | 1.7 | (1.1-2.5) | 0.01 |
| **Water storage containers in the yard** | | | | | |
| No | 516 | *57.4* | 1 |  |  |
| Yes | 183 | *72.7* | 1.8 | (1.2-2.9) | 0.01 |
| **Washing place in the yard** | | | | | |
| Yes | 53 | *49.1* | 1 |  |  |
| No | 646 | *62.4* | 1.9 | (0.9-3.7) | 0.07 |
| **Trees in the yard** | | | | | |
| Not any | 226 | *57.9* | 1 |  |  |
| 1 to 4 | 192 | *69.8* | 1.8 | (1.1-2.9) | 0.02 |
| ≥ 5 | 281 | *58.4* | 1.0 | (0.7-1.6) | 0.89 |
| **L3 + L4 + pupae of *Ae. aegypti*** | | | | | |
| Absence | 570 | *59.8* | 1 |  |  |
| Presence | 122 | *68.0* | 1.4 | (0.9-2.3) | 0.18 |
| **Number of *Ae. aegypti* larvae** | | | | | |
| 0 | 570 | *59.8* | 1 |  |  |
| 1 to 15 | 55 | *60.0* | 1.0 | (0.5-2.0) | 0.93 |
| ≥ 16 | 67 | *74.6* | 1.9 | (1.0-3.6) | 0.07 |
