# Supplementary material for: Evidence of Dengue Virus Transmission and Factors Associated with the Presence of Anti-Dengue Virus Antibodies in Humans in Three Major Towns in Cameroon
Source: PLoS Negl Trop Dis. 2014 Jul 10;8(7):e2950. doi: 10.1371/journal.pntd.0002950 (PMC4091864; doi:10.1371/journal.pntd.0002950)
Supplement: Table S3 — Sero-epidemiologic survey of dengue in Yaounde, Cameroon in 2006–2007: Univariate analysis using logistic regression with random effect. (DOC) [file pntd.0002950.s004.doc]

| **Risk factor** | **No. tested** | **% IgG** | **Univariate OR** | **OR 95%CI** | **p** |
| --- | --- | --- | --- | --- | --- |
| **Being born outside Yaounde** | | | | | |
| No | 352 | *6.8* | 1 |  |  |
| Yes | 252 | *11.9* | 1.9 | (1.0-3.4) | 0.04 |
| **History of travels outside Yaounde** | | | | | |
| Never | 81 | *4.9* | 1 |  |  |
| At least once | 526 | *10.5* | 2.2 | (0.8-6.4) | 0.13 |
| **Fever episode within the last 3 months** | | | | | |
| No | 365 | *7.9* | 1 |  |  |
| Yes | 242 | *12.4* | 1.6 | (0.9-2.8) | 0.07 |
| **Malaria access within the last 3 months** | | | | | |
| No | 441 | *7.7* | 1 |  |  |
| Yes | 166 | *15.1* | 2.1 | (1.2-3.7) | 0.01 |
| **Yellow fever vaccine within the last 3 months** | | | | | |
| Yes | 44 | *4.6* | 1 |  |  |
| No | 563 | *10.1* | 2.4 | (0.6-10.0) | 0.24 |
| **Having a fridge at home** | | | | | |
| Yes | 226 | *7.1* | 1 |  |  |
| No | 379 | *11.4* | 1.7 | (0.9-3.1) | 0.09 |
| **Having a car** | | | | | |
| No | 531 | *8.9* | 1 |  |  |
| Yes | 72 | *13.9* | 1.6 | (0.8-3.5) | 0.17 |
| **Stagnant water in the yard** | | | | | |
| No | 429 | *8.2* | 1 |  |  |
| Yes | 160 | *13.1* | 1.7 | (0.9-3.0) | 0.07 |
| **Green plants in the yard** | | | | | |
| No | 231 | *7.4* | 1 |  |  |
| Yes | 371 | *11.1* | 1.6 | (0.9-2.8) | 0.14 |
| **Any trees in the yard** | | | | | |
| 0 | 256 | *6.6* | 1 |  |  |
| 1 - 4 | 219 | *9.6* | 1.5 | (0.8-2.9) | 0.24 |
| ≥ 5 | 122 | *15.6* | 2.6 | (1.3-5.2) | 0.01 |
| **Banana trees in the yard** | | | | | |
| No | 443 | *7.5* | 1 |  |  |
| Yes | 154 | *16.2* | 2.4 | (1.4-4.4) | <0.01 |
